# Molecular Properties and Evaluation of Indion 234–Ondansetron Resinates

**DOI:** 10.4103/0975-1483.66799

**Published:** 2010

**Authors:** S Shah, S Pandya, MR Bhalekar

**Affiliations:** *Department of Pharmaceutical Technology, Pioneer Pharmacy Degree College, Vadodara, India*; 1 *Department of Pharmaceutics, AISSMS College of Pharmacy, Pune, India*

**Keywords:** Indion resin, ondansetron, taste masking

## Abstract

Ondansetron is a serotonin 5HT3 antagonist; anti-emetic drug. Bitter taste of the ondansetron is a major problem in ensuring patient compliance. The study was designed to formulate tasteless complexes of ondansetron with ion exchange resin and evaluate molecular properties of drug complex. The drug-loading process was carried out using various resins and was optimized using different drug:resin ratio and pH. Resinates were characterized by infrared spectroscopy, thermal analysis, and X-ray powder diffraction (XRPD). Indion 234 gave the best loading efficiency at drug resin ratio of 1:1.5. pH had no effect on drug loading. XRPD studies revealed that drug is in amorphous state in complex. The Infrared studies revealed complexation of secondary amine group of drug with carboxylic functional group of resin. Taste evaluation by using semiquantitative method found resonates as tasteless and agreeable. The release of drug from resinates in simulated gastric fluid was complete in 30 min. Thus, ion exchange resinates offer an effective tool for masking of bitterness and improve drug release.

## INTRODUCTION

Ion exchange resins are solid and suitably insolublilized high-molecular weight polyelectrolytes that can exchange their mobile ions of equal charge with the surrounding medium reversibly and stochiometrically.[1] The reaction is an equilibrium reaction.[2] Various articles describe the utility of ion exchange resins for taste masking, sustained release, targeted drug delivery, and drug stabilization.[3,4] The ion exchange resins are available in different size, cross linkage, and functionality making them suitable for various applications. Bitter cationic drugs can get adsorbed on to weak cationic exchange resin of carboxylic acid functionally to form the complex, which is nonbitter.[5] The taste-masking applications of ion exchange resins are reported for various cationic drugs, e.g., ciprofloxacin, chloroquin phosphate.[6,7] The ionic binding of the drugs to polymeric materials such as carbopol is emerging as an important mechanism of taste masking.[8] In this study, Indion resins were used to mask the taste of bitter drug ondansetron. Taste-masking ability of optimized complex, molecular properties, and physical characteristics were evaluated. The complex of cationic drug and weak cation exchange resin does not break at salivary pH (6–7) with cation concentration of 40 meq/L. However, at high cation concentration of stomach and pH of 1–3, free drug is immediately released.

## MATERIALS AND METHODS

### Materials

Ondansetron (Cipla Ltd., Mumbai, India), Indion 204, Indion 234, Indion 264 (Ion Exchange Ltd., Mumbai, India), and β- CD (Lupin Pharmaceutical, Pune, India) were obtained as gift samples. All other ingredients used throughout the study were of analytical grades and were used as received.

#### Ion exchange resin[9][Table 1]

**Table 1:** Ion exchange resin used

Resin	Functionality	Matrix	Form	Ion exchange capacity	Particle size
Indion 204	Carboxylic acid	Cross-linked polyacrylic	H^+^	10 meq/dry g min	+100 BSS: 1% max +200:45% max
Indion 234	Carboxylic acid	Cross-linked polyacrylic	K^+^	5.25 meq/dry g min	+100BSS: 1% max +200:30% max
Indion 264	Carboxylic acid	Cross-linked polyacrylic	H^+^	10 meq/dry g min	+100 BSS: 1% max +200:30% max

#### Ondansetron[10][Table 2]

**Table 2:** Ondansetron profile

Molecular weight	293.8
Bioavailability	Approximately 60%
p*Ka*	7.4
Elimination t_½_	4.1–11.6 h (oral) 2.5–6 h (IV)
Excretion	Renal
Solubility	Water soluble (10 mg/mL), insoluble in alcohol, acetone, methanol

#### Method of analysis

The drug was estimated spectrophotometrically at 272.6 nm using UV–VIS spectrophotometer (*V-520*, SR type, *JASCO* Corporation, Tokyo, Japan) over concentration range of 2-12 μg/mL.

### Optimization of drug-loading process

The resin that showed highest loading efficiency was optimized for the drug: resin ratio. The loading efficiency of optimized ratio was further checked to find optimum pH conditions for drug loading.

### Drug loading

For drug loading, batch method[11] was used, drug solution of concentration 1 mg/mL was prepared in deionized water. The required quantity of resin was placed in drug solution and was stirred till the attainment of equilibrium. Time for attainment of equilibrium was decided to be 6 h from preliminary experimentation. The slurry was filtered, and amount of drug remaining in the filtrate was determined spectrophotometrically.The amount of drug adsorbed was determined by the difference between amount of drug present in stock solution and amount remaining in filtrate at the end of equilibrium.

## EVALUATION OF RESINATES

### Determination of drug content[12]

Resinate (10 mg) was placed in 1 M HCl and stirred at 100 rpm for 1 h. The solution was filtered (Whatman filter paper no. 41) and analyzed for content of ondansetron. Stability of complexes was determined by placing weighed quantity of complex in deionized water for 24 h and analyzed for drug content.

### Taste evaluation[13,14]

Evaluation of taste was done in two parts.

#### Determination of threshold bitterness concentration

Various concentrations (1–30 mcg/mL) of drug were prepared in phosphate buffer pH 6.7. Mouth was rinsed with this solution and then, l0 mL of the most dilute solution was tasted by swirling it in the mouth mainly near the base of the tongue for 30 s. If the bitter sensation was no longer felt in the mouth after 30 s, the solution was spat out and wait for 1 min to ascertain whether this is due to delayed sensitivity. Then rinse with safe drinking water. The next highest concentration should not be tasted until at least 10 min have passed. The threshold bitter concentration is the lowest concentration at which a material continues to provoke a bitter sensation after 30 s. After the first series of tests, rinse the mouth thoroughly with safe drinking water until no bitter sensation remained. Wait for at least 10 min before carrying out the second test.

#### In vitro evaluation of bitter taste of resinates

An accurately weighed (8 mg drug equivalent) resinate and 10 mL of pH 6.7 phosphate buffer (0.1 M) was taken in series of volumetric flask and stirred at 50 rpm. The stirring was stopped at different time intervals such as 0, 10, 30, 60, and 120 s, dispersion was filtered, and the concentration of ondansetron in filtered resinate was determined. Time for resinate to achieve drug concentration corresponding to threshold bitterness in 10 mL phosphate buffer is recorded.[1]

### Micromeritic properties

Compressibility, angle of repose, and bulk density were determined using the methods described in the literature.[15]

### X-ray diffraction

X-ray diffraction patterns for ondansetron, Indion 234, resinate, and physical mixture of resin and drug were obtained on Phillips analytical X-ray BV (PW1710) (Almelo, the Netherlands) using Cu anode, 40 kV and current of 30 mA.

### IR diffraction

The Infrared spectra of ondansetron, Indion 234 and resinate were obtained using Fourier transform infrared (FTIR) spectroscopy (Jasco 460 plus, Tokyo, Japan) and spectra were observed over wave number 3600– 1000 cm^-1^.

### Differential scanning calorimetry study

Universal V4.1d TA differential scanning calorimeter (TA Instruments, USA) was used to analyze the thermal behavior of ondansetron, Indion 234, and ondansetron–Indion 234 complexes (resinate 234). Indium standard was used to calibrate the DSC temperature. The samples were heated at 10°C per minute.

### *In vitro* release of ondansetron

Weighed quantity of resinate equivalent to normal dose was subjected to dissolution studies using USP type II dissolution apparatus at 100 rpm with temperature of 37 ± 0.5°C and 900 mL 0.1 N HCl used as the dissolution medium. An aliquot equal to 5 mL was withdrawn at specific time interval, and it was filtered through Whatman filter paper no. 41. Absorption of the filtered solution was checked by UV spectroscopy at 272.6 nm and quantity of drug released was determined periodically(*n* = 5).

## RESULTS AND DISCUSSION

For the preparation of resinate, batch method was used because of its convenience.Table 3 shows that maximum amount of ondansetron binds to Indion 234 (drug content 46.30%) while Indion 204 and Indion 264 reported 36.96% and 42.88% drug content, respectively. This can be attributed to the difference of cross-linking, exchange capacity, and form of resin. The loading capacity of Indion 234 is a function of exchange of K^+^ ions in the resin with ions in solution.[15] Further attempt was made to optimize the drug-loading process by carrying out loading at different drug:resin concentration which showed 1:1 as the best ratio [Table 4]. An increase in amount of resin does not give correspondingly high increment in % drug content. It was seen that pH has no significant effect on drug loading [Table 5]. This can be explained as p*K*a of the drug is 7.4 which means the drug is ionized over entire pH range over which drug-binding studies are done while resin which has weak acid functionality (–COOH) will show better ionization at lower pH. Hence, pH of loading solution (3.5) was used for drug loading. Thus, resinate prepared by batch method using Indion 234 in drug:resin ratio of 1:1 at pH 3.5 gives optimum loading. The % drug content of optimized resinate was 44.75%. The drug content of resinate by dissolution was found to be 44.79%; the release of drug in deionized water was negligible (0.4%) which shows stability of complex in aqueous environment. Evaluation of taste was done using procedure described in WHO guidelines for determination bitterness value of bitter medicinal plant extracts and method described in the literature which is a fast and semiquantitative method and overcomes subjectiveness of time intensity method described in earlier literature.[16] Most volunteers reported the threshold bitterness at 20 μg/mL. The time for this threshold bitterness concentration to be achieved in buffer of salivary pH showed that the drug is not released in saliva to attain threshold bitterness concentrations thereby masking the bitter taste satisfactorily[Tables 6 and 7].

**Figure 1:**
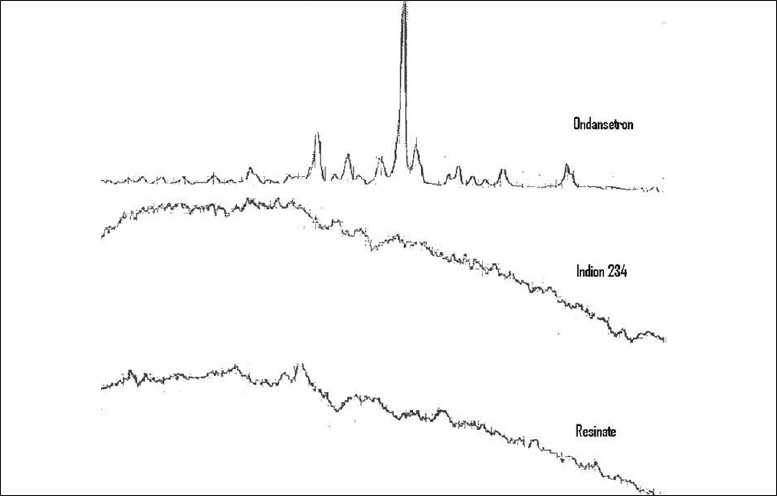
X-ray powder diffraction of ondansetron, Indion 234 and ondansetron—Indion 234 resinate

The complexation was confirmed by carrying out X-ray powder diffraction study on Indion 234, drug, and resinate. Ondansetron is crystalline whereas Indion 234 is amorphous resin. The molecular state of the drug in resinate shows hallow-diffused pattern and the absence of drug peaks.[6] This confirms that the entrapped drug is dispersed monomolecularly in the resin bead. The difference in peaks obtained at various θ values reveals that the samples are different from each other [Figure 1].

**Table 3:** Selection of resin (n = 3)

Resin	Drug:resin ratio	% Drug content of resinate
Indion 204	1:1	36.57 ± 0.4791
Indion 234	1:1	46.30 ± 0.3660
Indion 264	1:1	42.10 ± 0.3223

**Table 4:** Effect of drug:resin ratio on loading (*n* = 3)

Resin	Drug:resin ratio	% Drug content of resinate
Indion 234	1:0.5	58.02 ± 0.4083
	1:1	46.30 ± 0.3885
	1:1.5	42.10 ± 0.3223
	1:2	32.20 ± 0.2405

**Table 5:** Effect of pH on loading (n = 3)

Resin	Ratio	pH	% Drug content of resinate
Indion 234	1: 1	2	41.26 ± 0.2567
		3	43.18 ± 0.4718
		3.5	44.45 ± 0.376
		5	45.43 ± 0.379
		6	46.17 ± 0.3325
		7	44.68 ± 0.3687

**Table 6:** Determination of threshold bitterness concentration

Volunteers	1	2	3	4	5	6	7
Threshold bitterness concentration (μg/mL)	10	10	20	20	20	20	20

**Table 7:** Time for attainment of threshold bitterness concentration *in vitro* (n = 5)

Stirring time (s)	0	5	10	30	60	120
Concentration (μg/mL)	1.25 ± 0.124	1.45 ± 0.2587	1.57 ± 0.2478	1.91 ± 0.1569	3.75 ± 0.1782	6.62 ± 0.2247

**Figure 2:**
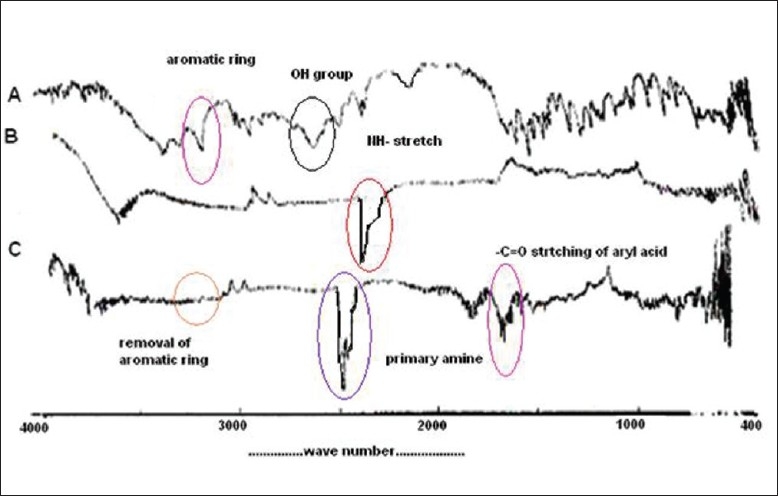
Infrared spectra of (A) ondansetron, (B) Indion 234 and (C) ondansetron—Indion 234 resinate.

**Figure 3:**
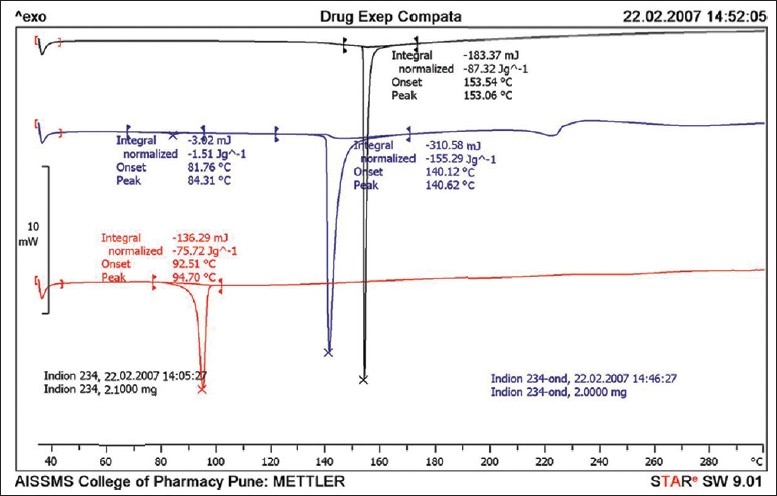
Differential scanning thermograms ondansetron, Indion 234 and ondansetron—Indion 234 resinate

The IR spectra of ondansetron, Indion 234, and resinate are depicted in Figure 2. Drug spectrum shows a prominent peak at 3320.82 cm^-1^ corresponding to the –NH_2_ stretching in a secondary amine. Aromatic ring and carbonyl group also present in drug interaction. Indion 234 shows characteristic peaks at 1674 cm^-1^ corresponding to –C=O stretching of aryl acids and at 1602 cm^-1^ due to aromatic C=C stretching. The absence of peak (3320.82 cm^-1^) in IR spectrum of resinates confirms complexation of the secondary amine group in the drug with resin [Figure 3].

The thermal behavior of the pure drug shows endotherm at 181.16 and 244.10°C corresponding to melting and degradation. Indion 234 shows endotherm near 126° and 298°C whereas resinate shows endotherm near 254°C thus indicating complexation.

**Figure 4:**
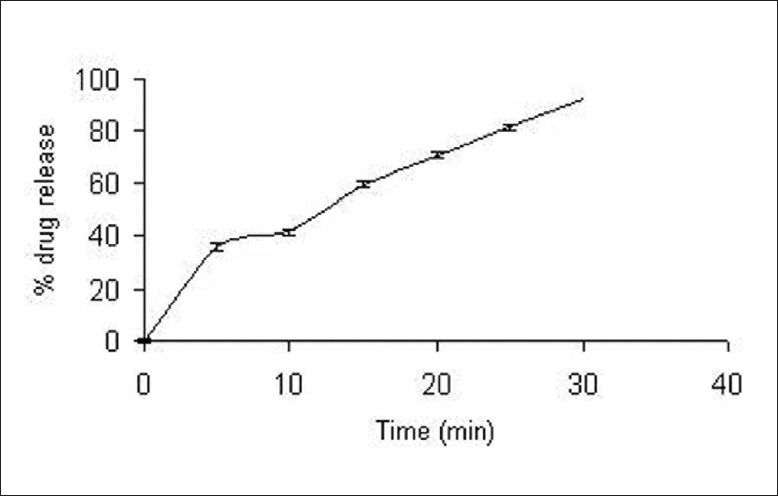
*In vitro* drug release from Indion 234-ondansetron resinate

The complex was subjected to dissolution studying 0.1 N HCl using USP 23, paddle apparatus at 100 rpm at 37 ± 1°C shown that drug release was more that 80%[Figure 4].

Further, the micromeritic properties were evaluated using properties such as bulk density, angle of repose, and compressibility (Carr) index are calculated in Table 8. It was found that all the properties of resin and resinate were similar and the flow and compressibility of resinates are satisfactory.

**Table 8:** Micromeritic properties of resin 234 and resinate (n = 3)

Property	Drug	Resinate
Carr index	19.86%	21.12%
Bulk density	0.3125 g/mL	0.377 g/mL
Angle of repose	27.89	28.24

## CONCLUSION

This incorporates the study on formulation of tasteless complexes of ondansetron with ion exchange resin and evaluation of molecular properties of drug complex. Drug loading was performed using various resins and was optimized using different drug:resin ratio and pH. Drug–resin complexes were characterized by infrared spectroscopy, thermal analysis, and X-ray powder diffraction (XRPD). Indion 234 gave best-loading efficiency, and it was also reported that pH had no effect on drug loading. It was revealed by XRPD studies that drug was in amorphous state in complex and the IR studies revealed complexation of secondary amine group of drug with carboxylic functional group of resin. Taste evaluation done by semiquantitative method established that resonates were tasteless and agreeable. The release of drug from resinates in simulated gastric fluid was complete in 30 min. Thus, it was shown that ion exchange resinates can be effectively used for masking bitter taste and also to improve drug release.
